# Annual Announcement of the Med. Faculty of McGill Col. Montreal

**Published:** 1851-10

**Authors:** 


*Annual Announcement of the Medical Faculty of McGill College, Montreal, for Session* 1851–2.

The system of medical education pursued in the McGill College of Montreal, differs materialy from that of similar institutions in the United States. Each session continues six months, and each candidate for graduation is required to attend four courses before he can present himself for examination for the degree of Doctor of Medicine and Surgery. He is also required to give proof of having attended the practice of the Montreal, or some other hospital, having beds for at least forty patients. He must moreover furnish evidence of competent classical attainments.

The Faculty consists of one professor, nine lecturers and one demonstrator. Each teacher is required to deliver at least five lectures during the week; each of one hour's duration, and to give the pupils one examination, which, however, is regarded as equivalent to one lecture. The departments in the institution are, Anatomy and Physiology; Chemistry;

Theory and Practice of Medicine; Midwifery and Diseases of Women and Children : Principles and Practice of Surgery; Materia Medica and Pharmacy; Clinical Medicine; Clinical Surgery; Practical Anatomy; Institutes of Medicine; Forensic Medicine, and Botany.

The medical degree granted by McGill College is that of Doctor of Medicine and Surgery.

